# Cost-effectiveness of current approaches in rectal surgery

**DOI:** 10.1016/j.amsu.2019.07.004

**Published:** 2019-07-07

**Authors:** Khalid N. Alsowaina, Christopher M. Schlachta, Nawar A. Alkhamesi

**Affiliations:** a Canadian Surgical Technologies & Advanced Robotics (CSTAR), London Health Sciences Centre, Canada; b Department of Surgery, Western University, London, Ontario, Canada

**Keywords:** Robotics, Laparoscopy, Cost-effectiveness, TaTME, Rectal surgery, Colorectal

## Abstract

Colorectal cancer is ranked as the fourth malignant cause of mortality. With the tremendous revolution in the modern medical techniques, minimally invasive approaches have been incorporated into rectal surgery. The effectiveness of surgical procedures is usually measured by a combination of qualitative (quality of life) and quantitative (years of life) measures, while the costs should reflect the use of different resources that were involved in delivering the medical care and they are affected by several factors, including length of hospital stay. In this review, we provide an insight into the cost-effectiveness of the different types of rectal surgeries in order to present a systematic approach for future preferences. A comprehensive literature review using Medline (via PUBMED), Embase and Cochrane Central Register of clinical trials (via clinical trial.org) was performed. Minimally invasive rectal surgeries have considerable cost-effective properties that outweigh those of the open techniques in terms of earlier return to bowel function, lower morbidity rates, reduced pain, shorter length of hospital stay and the overall patients’ quality of life although there was no difference in long-term oncological and survival outcomes. The paucity of currently available long-term oncologic, quality of life, and economic outcomes may limit an adequate comparison of robotic surgeries to other surgical techniques. It is therefore recommended to conduct focused studies to help balance the cost/benefit factors along with other technical considerations aimed at reducing the cost of robotic systems with subsequent improvement of their cost-effectiveness.

## Highlights

• Colorectal cancer is the fourth cause of mortality. Minimally invasive surgery is now considered the standard of care.
• Cost of minimally invasive surgery is offset by the better outcomes due to less complications and shorter hospital stay.
• TaTME requires two working teams and two sets of instruments which increases the total cost.
• Robotic surgery in obese patients demonstrated superior results when it comes to hospital stay and overall complications.
• Well conducted clinical trials looking at cost effectiveness of new technologies in colorectal surgery are needed.

## 1 Introduction

Colorectal cancer is ranked as the fourth malignant cause of mortality as it is associated with about 700,000 deaths on the global level each year [1]. With the exception of the most developed countries, both the newly reported cases of colorectal cancer and the relevant mortalities are still progressively increasing [2]. During the 1980s, open total mesorectal excision (TME) has been the standard procedure for the treatment of rectal cancer [3]. Later, a significant shift from open procedures to laparoscopic techniques led to attaining a minimally-invasive approach that contributed to obtaining promising outcomes in selected patients [4]. Due to its technical demands in patients with low rectal cancer, the anatomical considerations in male patients, and the probability of conversion to open approaches due to limited visualization of the distal tumor margins, it has been imperative to find suitable alternatives to the laparoscopic total mesorectal excision (LaTME) procedure. In this context, transanal TME (TaTME) has emerged and provided reliable outcomes on the short- and long-term scales [5,6]. With the tremendous revolution in the modern medical techniques, robotics has been incorporated into the surgical platforms as a minimally-invasive technique that yields nearly no minimal human error. However, new surgeon generations are required to adopt the novel technique and the outcome of the procedure should match or outweigh its costs [7]. The latter issue must be considered, particularly with the parallel burden of limited human resources, lack of access to the basic infrastructure in low-income and some middle-income countries, and the increasing healthcare costs for different surgeries. Since approximately one-third of the global health spending is related to surgeries [8], it is important to deliver these life-saving approaches in a cost-effective manner.

The effectiveness of surgical procedures is usually measured by a combination of qualitative (quality of life) and quantitative (years of life) measures, while the costs should reflect the use of different resources that were involved in delivering the medical care and they are affected by several factors, such as the length of hospital stay [9]. In general, the balance of an intervention effectiveness for the patients, taking into account their future quality of life, and the cost incurred by the patient and society is important also for rectal surgical procedures since they are usually associated with high equipment cost, especially the new approaches. In the present review, we provided an insight into the cost-effectiveness of the different types of rectal surgeries in order to present a systematic approach for future preferences.

## 2 Methods

A comprehensive literature review using Medline (via PUBMED), Embase and Cochrane Central Register of clinical trials (via clinical trial.org) was performed. To increase the sensitivity of identifying all relevant articles, the following medical subject headings (MeSH) terms used in combination using the Boolean operators. There were no limits for language or publication date since it has been assumed that the articles that compare the aspects pertinent to the cost-effectiveness of different rectal surgery techniques would be relatively scarce. The used keywords were as follow: “open rectal surgery”, “laparoscopic rectal surgery”, “Transanal total mesorectal excision”, “robotic rectal surgery”, “open rectal surgery AND Transanal total mesorectal excision”, “open rectal surgery AND laparoscopic”, “open rectal surgery AND robotic”, “laparoscopic rectal surgery AND robotic”, “laparoscopic rectal surgery AND Transanal total mesorectal excision”. The keyword “cost-effectiveness” was utilized whenever possible. The “advanced search” utility was used in the medical database to place the important keywords as appropriate. Further, we performed a meticulous checking of the references of the included articles to find suitable comparative evidence. Only articles that have been published in peer-reviewed journals were considered for inclusion in the present review.

### 2.1 Open vs. laparoscopic rectal surgery

Open rectal surgery (ORS) was effectively considered the primary surgical approach for the treatment of rectal cancer. However, laparoscopic surgery (LRS) provided a less-invasive approach that is associated with lower morbidity when compared to the open procedures. The overall benefits for the patients as well as the cost of both ORS and LRS were formerly investigated in several studies. King et al. [10] recruited a relatively small number of patients (n = 62) experiencing rectal adenocarcinoma for a follow-up period of 3 months. Patients who underwent LRS had a significant reduction of the length of hospital stay (LOS) (p < 0.018) and the total postoperative, convalescent, and readmission stay (p < 0.012) as compared to ORS. However, the total costs of LRS were higher. Regarding postoperative effectiveness, there was no difference in the quality of life indicators at the end of the follow-up period. The total cost of laparoscopic surgery was 6433.4 British Pounds in comparison to 6786.9 in the open group, making the laparoscopic surgery the favorable option.

For a longer follow-up period (mean 53.6 months), Braga et al. [11] conducted a randomized controlled trial on patients with low or middle rectal cancer who underwent ORS (n = 85) and LRS (n = 83) and studied the postoperative health and financial outcomes. Although there was no difference in the postoperative morbidity rates between both patient groups, LRS was associated with significantly shorter LOS (P = 0.004), better quality of life during the first year following surgery (P < 0.0001), with a slight increase in the net hospital costs when compared to ORS surgeries (351 American Dollars), this difference in cost was due to additional OR charges in the laparoscopic group. The author also explained that post-operative complications cost extra 1396 American Dollars in the open group in comparison to the laparoscopic group.

Similar results were observed by Zhou et al. [12]. In this study, the patients follow up period was 56.6 months where no significant difference in the rates of recurrence, overall survival and disease-free rates following surgical intervention. However, the laparoscopic group exhibited rapid recovery and shorter LOS, whereas their mean operative time was longer and with higher direct costs (5532 vs. 3913 American Dollars). It is worth noting that the author classified the open group as minilaparotomy (skin incision ≤ 7 cm in length below the umbilicus) and informed us that they did not perform splenic flexure mobilization in the open group because of small incision unlike the laparoscopic group which required splenic flexure mobilization in some cases. Unfortunately, the authors did not explain what happened to the cases in the open group when the splenic flexure mobilization was required. This type of information is important in our opinion to evaluate this paper appropriately and it will affect the outcome and the cost associated with it.

On the other hand, a more recent case-matched study showed relatively different results. Keller et al. [13] found that postoperative complications, rate of readmission, and reoperation were similar following ORS and LRS. Additionally, ORS was associated with a significant increase in the operative time and operative costs, higher total hospital costs, and longer LOS. Further, at discharge, patients in the ORS group required home care and skilled nursing services, indicating that their quality of life was affected. Nonetheless, matching the patients in the ORS to their counterparts according to the age, tumor distance, and BMI may limit the results. The reduced LOS after LRS might be attributable to less operative bleeding, reduction of postoperative complications, early recovery of oral feeding and bowel functionality, and decreased postoperative pain (less analgesics) [14]. Postoperative quality of life is less affected after LRS as the laparoscopic technology augments the rate of anal preservation [15]. The overall increased cost of LRS operations, as demonstrated by additional costs of surgical instruments and longer operative time, might be compensated by the short LOS and lower postoperative costs, such as reoperations, postoperative complications, rates of utilizing intensive care units, and requiring advanced home nursing care [10,13]. Additionally, the number of days where the patients would take off days in paid work was reduced after LRS, which could be added to the aforementioned compensatory factors [10]. That leads to making laparoscopic surgery the most cost-effective approach.

### 2.2 Transanal total mesorectal excision vs. laparoscopic rectal surgery

The TaTME technique seems to be a feasible and safe procedure for the treatment of rectal cancer as it is has several advantages, particularly in regards to make the vision and dissection easier due to the use of carbon dioxide from the perineal approach in addition to abdominal insufflation [16]. To the best of our knowledge, the actual cost-effectiveness of TaTME surgeries was not directly studied or compared to other techniques. However, the efficacy of this technique can be concluded from both the short- and long-term outcomes in the comparative studies. Short-term outcomes (for 1 month) revealed that TaTME was associated with shorter intraoperative time when performed by two surgical teams and less frequent early readmissions, while the achievement of oncological resection principles was not different when compared to the traditional LRS [17]. Upon comparing patients’ specimens, Velthuis et al. [18] demonstrated that complete mesorectum was achieved in 96% of the patients following TaTME procedures, compared to 72% in the LRS group. Although the promising outcomes of TaTME, it is important to consider that the technique is performed by two teams working simultaneously, which requires more surgical equipment, more staff, and more trained personnel, increasing the possibility of yielding high costs. This issue could be resolved when the anticipated North American TaTME Trial conclude.

### 2.3 Robotic vs. laparoscopic rectal surgery

With the widespread usage of LRS, some limitations and technical considerations have emerged, such as the two-dimensional view, poor ergonomics, and the fulcrum effect that yielded several challenges during performing the manual surgical maneuvers during the procedure [19]. Robotic rectal surgeries (RRS) provide a more convenient approach with a high degree of dexterity due to the magnified view. This led to a rapid adoption of the technique during the past decade in the United States and Europe [20]. However, the major questionable drawback of RRS is its high cost, which should be matched to its benefits for the patient and operating surgeons.

RRS was consistently associated with higher costs than LRS procedures [[21], [22], [23]]. In addition to the initial investment of the robotic system by the healthcare institution, maintenance costs, consumables, and longer operative times were the significant contributing factors. For example, Halabi et al. [24] performed an analysis of RRS- and LRS-related outcomes in the United States and found that the yearly maintenance of the robotic system might require approximately $100,000, increasing the total hospital charges significantly following RRS (odds ratio $12,964.90; 95% CI 6,534.79–19,395.01). Further, it has been demonstrated that the use of robotic platform might add an average $5000 for every patient, increasing the net cost by 1.5 times due to using the *da Vinci* robot platform [22,23]. Similarly, in other studies, RRS was associated with a significant increase in the total and procedure-related costs in patients with rectal cancer when compared to LRS where the cost of RRS was 2.4 times higher than LRS [21,25]. It is worthy to note that the increase in RRS cost can be partially compensated by increasing the number of daily performed operations in highly specialized medical institutions, which could be assisted by the short operative times, reduced LOS, and decreased morbidities [20]. Morelli et al. [26] suggested exerting future efforts to reduce the fixed robotic costs, such as the amortized costs of the robot and laparoscopic instruments as well as the purchase and maintenance costs.

Additionally, longer intraoperative times in RRS are caused by docking and repositioning of the ports, especially in multiquadrant rectal cases [24]. With more experience acquisition and modification of surgical techniques, it seems that the total consumed time its relevant charge will be reduced [27,28]. Indeed, this is consistent with the results of Morelli et al. [26], where they observed that the median operative time was significantly reduced with the progressive shift of the learning curve from an initial learning period to a more complicated phase with the robotic experience and technical competence. Also, with the introduction of new generations of robots which allowed single docking for multiquadrant rectal surgery, significant reduction in time has been observed with increase OR time efficiency.

It is necessary to note that the rate of conversion to open surgeries was significantly low in RRS as compared to LRS as per reported in 2009 in patients with rectal cancer [29]. Similarly, Halabi et al. [24] found a significant reduction in the conversion rate following robotic procedures with an odds ratio 0.10 (confidence interval 0.06, 0.16) when compared to LRS. Actually, this property is vital as the conversion to open surgery is substantially associated with higher costs, longer LOS, and higher morbidity and mortality [30,31]. This might be related to allowing better visualization, ergonomics, and higher degrees of freedom during performing RRS rather than LRS, suggesting that RRS is superior for the treatment of lower rectal tumors [29,32].

Other operative and postoperative outcomes were comparable between the LRS and RRS. For example, intraoperative blood loss was not significantly different during both techniques in patients having rectal cancer as shown by Morelli et al. [26] and Baek et al. [22]. However, it has been shown that RRS was associated with a lower number of hemorrhagic complications due to better visualization, easier dissection and suturing when compared to LRS [33]. From another perspective, several studies have shown that the LOS was similar following LRS and RRS surgeries [21,22,25] which ranged between 4 and 11 days [24,26]. This observation can confirm that the LOS is not relevant to the observed increase in total costs following RRS. Considering obese patients, RRS led to a significantly shorter LOS, and lower 30-day readmission rate although it the operative time was longer with higher intraoperative costs when compared to LRS [34,35]. The overall oncological outcomes [36] and postoperative quality of life [37] were not different in the patients after undergoing LRS and RRS.

### 2.4 Open vs. laparoscopic vs. robotic rectal surgery

In 2015, the clinical data of the patients who underwent ORS, LRS, and RRS to perform abdominoperineal resection were investigated over 3 years [33]. Regarding the operational effectiveness, it has been found that the minimally invasive procedures (LRS and RRS) were associated with lower morbidity risks, including urinary tract infection, hemorrhagic complications, and paralytic ileus, as compared with ORS. However, there was no difference in mortality between all groups. Although the total cost of RRS procedures were significantly higher that other surgeries, the overall outcomes of both RRS and LRS were better than ORS. When compared to both LRS and ORS, robotic surgeries had the lowest hemorrhagic complication rates, which is a consistently observed criterion of RRS [33].

A more recent retrospective study of rectal cancer surgeries [23] during a 7-year period compared the pathological and economic outcomes following ORS, LRS, and RRS. Patients in the RRS group had lower rates of conversion to open surgeries, and lower intraoperative hemorrhage as compared to LRS and ORS. The operative time was significantly longer in the robotic approach due to docking and undocking as well as the low degree of experience with the system. Total costs of RRS were significantly higher than ORS and LRS, which could be attributable to the costs of the operative room, post anesthetic care unit, and laboratory charges [23]. The median of post anesthetic care unit (PACU) charges were 619.66 Canadian Dollars, 464.47 and 583.93 for open, laparoscopic and robotic respectively. The median of laboratory charges were 1015.96 Canadian Dollars, 1032.16 and 567.67 for open, laparoscopic and robotic respectively [23].

## 3 Conclusion

Minimally invasive rectal surgeries have considerable cost-effective properties that outweigh those of the open surgery in terms of earlier regaining bowel function, lower morbidity rates, reduced pain, shorter length of hospital stay and the overall patients’ quality of life. The cost-effectiveness of robotic system appears to be more promising on the short-term period with similar long term outcomes to laparoscopic surgery. However, the cost of robot-assisted surgery is generally higher by 1.5–2.4 times per patient in comparison to laparoscopic surgery. Therefore, the paucity of currently available long-term oncologic, quality of life, and economic outcomes may limit an adequate comparison of robotic surgery to other surgical techniques. It is therefore we recommend further controlled clinical trials, employing experienced surgeons, to help balance the cost/benefit factors along with other technical considerations aimed at reducing the cost of new technologies in the management of rectal diseases with subsequent improvement of their cost-effectiveness.

## Provenance and peer review

Not commissioned, internally reviewed.

## Ethical approval

Not applicable.

## Sources of funding

No financial support and sponsorship.

## Author contribution

Khalid N. Alsowaina: Data gathering, design and manuscript writing.

Christopher M. Schlachta: Data and manuscript review.

Nawar A. Alkhamesi: Manuscript design, data review and final approval.

## Conflicts of interest

All authors declared that there are no conflicts of interest.

## Trial registry number

Not applicable.

## Guarantor

Nawar A. Alkhamesi MD, PhD, FRCS (Gen. Surg.), FRCS, FRCSEd, FRCSC, FACS, FASCRS.

Canadian Surgical Technologies & Advanced Robotics (CSTAR)

University Hospital, London Health Sciences Centre.

339 Windermere Road.

London, ON, CANADA.

N6A 5A5.

T: 1-519-663-3985.

F: 1- 519-663-3052.

E: nalkham2@uwo.ca.
